# Evaluating Adolescent Patient Outcomes and Staff Member Injuries at a General Psychiatric Inpatient Unit

**DOI:** 10.3390/bs14090737

**Published:** 2024-08-24

**Authors:** Patrick W. Romani, Ava Anjom, Tyler Anderson, Merlin Ariefdjohan

**Affiliations:** 1 Department of Psychiatry, School of Medicine, University of Colorado, Anschutz Medical Campus, Aurora, CO 80045, USA; patrick.w.romani@ucdenver.edu (P.W.R.); ava.anjom@utah.edu (A.A.); 2 Department of Counseling Psychology, University of Northern Colorado, Greeley, CO 80639, USA; tyler.j.anderson@cuanschutz.edu

**Keywords:** dialectical behavior therapy (DBT), psychiatric inpatient unit, psychological assessment, staff member injury

## Abstract

Short-term pediatric psychiatric hospitalization is used to manage acute-crisis behaviors. Few studies have detailed their clinical model and key metrics such as patient behavioral health outcomes and staff experience. This study describes a model which emphasizes group therapy based on dialectical behavior therapy during brief inpatient stays (average length of stay of 8 days). The study variables assessed included patient symptoms of depression, anxiety, and anger at discharge, patient satisfaction, and staff safety. The program produced significant improvements in adolescent depression, anxiety, and anger, and patients reported high satisfaction with the services received. However, there was a high rate of staff injuries, correlated with staffing ratios and the time of day. The key findings from this study demonstrate the effectiveness of brief inpatient programs and highlight variables that may impact staff experiences on these units, which could serve as further discussion points to improve clinical care.

## 1. Introduction

There is a high prevalence of psychiatric disorders among children and adolescents globally, with an estimated number of cases reaching approximately 7.7 million in 2016 in the United States alone [1]. For some of these individuals, emergent psychiatric services will be necessary. Psychiatric hospitalization is essential for establishing safety and stability when these individuals experience an acute episode of psychiatric crisis. Psychiatric crises might include suicidal ideation, homicidal tendencies, and out-of-control behavior [2,3,4,5,6]. Over the past 20 years, research has documented a 25.8% increased use of psychiatric hospitalization for youth, with depressive disorders and suicidal ideation accounting for almost half of all psychiatric admissions [3,7]. In sum, psychiatric hospitalization is an essential and highly utilized mode of treatment for youth experiencing a psychiatric crisis.

While necessary, treatment at psychiatric inpatient units (PIUs) is costly, which contributes to the large economic burden associated with treatment of mental health disorders [3,8,9,10]. In trying to find a compromise between the cost of hospitalization and its benefits toward patient recovery, the average length of stay in an inpatient unit has been reduced from an estimated 12.8 days to approximately 7–9.6 days between 2006 and 2012 [11,12]. Thus, PIU providers are tasked with managing the most challenging mental health problems in a short amount of time. In addition to treatment constraints, there is a paucity of research on effective treatment modalities to use on PIUs. The arguably most researched modality of group-based treatment delivered at PIUs is grounded in the principles of dialectical behavior therapy (DBT) [13,14]. DBT is an evidence-based treatment for youth suicidal ideation, aggression, and personality disorders [15,16]. Using the biosocial theory, patients learn how biology and their environment can combine to produce patterns of behavior that do not align with their future goals. Patients receiving DBT learn strategies related to emotional regulation, distress tolerance, mindfulness, and interpersonal effectiveness to accept the difficulties in their life and work to solve those difficulties using healthy strategies. Given the short lengths of stay in PIUs, most units incorporate aspects of DBT into their group-based treatments. To evaluate the impact of integrating DBT at a PIU, one research group compared a “treatment as usual” group to a DBT treatment group [13]. These researchers showed significantly lower self-reported symptoms of depression following inpatient treatment with DBT. Other researchers have documented similar findings [14]. Thus, it seems that group-based treatment that incorporates aspects of DBT reduces self-reported symptoms of depression, anxiety, or anger at PIUs.

While patient symptoms gradually improved over their PIU admission, direct-care staff providing most of the treatment needed to manage potential assaultive behaviors, like aggression or self-injury [17]. Managing these assaultive behaviors involve risky procedures like restraint and seclusion, which have been shown to significantly relate to staff member injury occurrence [18,19]. Staff member injuries represent a complex problem for hospital settings [20]. Injuries affect staff morale, staff retention, and can be quite expensive [21,22]. For example, one study showed that staff injuries accounted for 2% of one hospital’s budget [17]. A growing evidence base of research shows staffing patterns [23], use of behavior management programs [19,24], comprehensive staff training programs [25], use of protective equipment [26], and being mindful of patient diagnosis [27] relates to staff member safety. However, most of this research comes from adult PIUs. There needs to be more research understanding the prevalence and potential causes of injuries at pediatric PIUs with evidence-based therapeutic programs already integrated.

In summary, implementing group therapy based on DBT principles in a PIU leads to positive patient outcomes. However, staff injuries at PIUs remain unacceptably high. Limited research exists on the occurrence and the situations that lead to these injuries within clinical care settings that implement DBT. Thus, this study has two objectives. First, the study aimed to describe a PIU treatment model and its influence on patient-reported symptoms of depression, anxiety, and anger. Second, the study evaluated the situations that led to staff injuries over a 1-year period. Overall, we hope that the key findings from this study will provide insight into patient outcomes and factors related to staff injuries, while also offering another description of PIU care models that might be applicable elsewhere.

## 2. Methods

### 2.1. Participants and Setting

The PIU selected for this study operated within an urban academic medical center providing specialized psychiatric care for adolescents experiencing psychiatric crises (e.g., following a suicide attempt). Clinical procedures took place at an 18-bed inpatient unit. Upon admission to the PIU, the nursing staff assigned each patient to their own bedroom. Common programming areas included three classrooms (approximately 20 to 30 m^2^ in size housing two tables and at least 10 chairs per classroom) and a cafeteria. Unit staff divided the classrooms by the age of the patients. The PIU worked with youth between the ages of 12 and 14 years old (i.e., tweens), and adolescents between the ages of 15 and 17 years. Patients averaged 15.4 years of age. Please refer to Table 1 and Table 2 for additional demographic information about the patient population served at this PIU. The PIU was staffed by university faculty and hospital staff. The university faculty consisted of three psychiatrists and a psychologist. The hospital staff consisted of 45 direct-care staff (holders of bachelor’s or master’s degrees in psychology or related field), 10 nurses (holders of bachelor’s degree in nursing), and three licensed behavioral health clinicians (master’s degree in professional counseling or social work). The dataset for this study included comprehensive data from all patients who were being treated at the PIU during the study period (July 2020 to December 2021), and corresponding data related to staff injury. Such data were already collected for clinical purposes. All data were de-identified to ensure confidentiality prior to being shared with the authors for analysis. The protocol for this retrospective study was approved by the authors’ institutional review board.

### 2.2. Dependent Variables

**Patient Outcomes.** Patients receiving treatment at the PIU during the study period were asked to complete the Anger, Depression, and Anxiety Scales from the Patient-Reported Outcomes Measurement Information System (PROMIS). Patients responded to questions structured according to a 5-point ordinal scale about whether the item was never, rarely, sometimes, often, or always true in the past 7 days [28]. The anger scale measured angry moods, verbal aggression, and physical aggression [29]. The depression scale measured symptoms of depression, such as feeling sad [28]. Finally, the anxiety scale measured fear, anxious misery, hyperarousal, and somatic symptoms related to arousal [30]. A previous study indicated alpha-coefficient values of 0.90, 0.95, and 0.93 for the anger, depression, and anxiety scales, respectively, which collectively reflects a high degree of assessment reliability [31]. An additional study by the same author [32] indicated a strong convergent validity of the PROMIS anger, depression, and anxiety measures with other established measures of these domains of emotions [31,32]. These outcome measures were administered upon admission to the PIU and at discharge (either the night before or the morning of patients’ departure from the unit). Patients completed the Patient Satisfaction Inventory at the conclusion of their admission. The authors created this inventory to measure patient satisfaction with medication management and the therapy services received, as well as whether they felt that the skills learned during their admission would be helpful outside of the hospital. This inventory consisted of a 1–7 Likert-type scale in which scores of 1 represented low satisfaction and scores of 7 indicated high satisfaction.

Patients completed these questionnaires using a tablet computer as overseen by the unit psychologist or a psychology trainee. Assistance was provided if the patient struggled to read or reported being unable to understand the question.

**Staff Member Injury.** The research team extracted pertinent data from the daily records collected by the Occupational Health Services department of the study site and from the clinic registry. These data included the number of staff and patients working on the unit throughout each day, frequency of restraint or seclusion usage, the number of staff member injuries, and the time each staff member injury occurred. We defined a staff member injury as any incident reported to the Occupational Safety and Health Administration (OSHA) by PIU-employed staff that is caused by patients who were already admitted to the PIU. This includes minor injuries such as bruises or scratches, as well as more serious incidents like concussions. Additional demographic information about the patients who injured the staff was gathered from the electronic medical record at the study site.

Considering the complexity of the data extraction process, data entry integrity was ensured by having a second, independent observer auditing the work of the primary data coder. This second observer input data on each of the variables described above for 30% of days across each month of the study period. We defined exact agreement as when the primary data coder reported the same value for the dependent variables described above (e.g., number of restraints used each day) as the secondary data coder. A disagreement was defined as when the primary data coder reported a different value than the secondary data coder for the variables described above. The research team calculated agreement by dividing the number of agreements by the number of agreements plus disagreements and multiplying this quotient by 100 to produce a percentage. The agreement between the two observers was 100%. 

### 2.3. Procedures

The PIU delivered interdisciplinary treatment by a child and adolescent psychiatrist, social worker, and psychologist. The psychiatrist evaluated each patient’s medication regimen as well as assisted with diagnostic conceptualization. The psychiatry team collaborated closely with the rest of the interdisciplinary team to adjust medications based on patient response to intervention. The social worker provided family therapy and assisted with identifying after-care services for the families. The psychologist organized the DBT treatment each patient received on the unit. The social worker and psychologist closely collaborated to ensure that the DBT material contacted by the patients could be integrated into the family therapy services.

Prior to starting data collection on patient outcomes, the unit’s psychologist and social workers trained PIU staff to deliver the DBT groups. The training lasted 2 h and consisted of a didactic presentation and role-play with coaching and feedback. The didactic presentation began by describing the history of DBT along with a description of the groups the unit would be conducting with patients (30 min). After this, the psychologist and social workers modeled one of the therapeutic groups (15 min). The trainers then provided all staff with a protocol of one of the DBT groups and divided them into groups of four. PIU staff took turns implementing the group according to the protocol, while either the psychologist or social workers provided feedback and support (60 min). At the end of the coaching period, the psychologist or social workers encouraged PIU staff to ask additional questions (15 min).

DBT can have a lengthy course of implementation, which is not feasible for short-term PIU admissions. Thus, DBT programming at the study site was modified to selectively focus on learning about mindfulness skills, distress tolerance, and emotional regulation. The unit schedule incorporated one DBT group, a group led by an occupational therapist, and snack/meals during the morning. After lunch, patients were visited by their families and then participated in a DBT and social skills group. Free time and snacks/meals were also scheduled as part of afternoon activities. During the evening, patients ate meals, visited with their families, and engaged in a wrap-up group that reviewed programming topics for the day. The Tween and Adolescent groups engaged in the same activities but within their respective age groups, meaning patients in the Tween group did not participate in programs with those in the Adolescent group.

Patients began each group session by participating in a mindfulness activity (e.g., mindful eating, mindful exercise, and mindful coloring) as led by the PIU staff. Afterward, PIU staff conducted the assigned DBT group. The unit conducted DBT groups according to a weekly schedule. On Mondays, the groups focused on introducing mindfulness and DBT to patients. Patients learned about dialectics, the biosocial theory, the three mind states, and how to use mindfulness strategies. Tuesdays and Fridays involved discussion of distress, identification of personally distressing situations, and formulation of coping strategies to manage distress using the six senses (e.g., vision, hearing, smell) or other distraction strategies. More specifically, patients learned about “Wise Mind ACCEPTS” (i.e., activities, contributing, comparisons, emotions, pushing away, thoughts, and sensations) and practiced using distraction or self-soothing activities based on the six senses during the group. On Wednesdays and Sundays, staff facilitated discussions on emotion regulation, including distinguishing negative emotions and antecedents to these emotions, recognizing positive attributes or experiences, and identifying and preparing to use various coping strategies. Example activities covered during the groups included discussions about accumulating positive experiences with opportunities to practice tracking positive experiences to “build a life worth living.” Finally, on Thursdays and Saturdays, the PIU staff reviewed dialectics to discern the existence of multiple truths to a situation as a way to reinforce positive behaviors and decrease maladaptive behaviors. Patients learned how to think and act dialectically during these groups. They reviewed example scenarios of triggering situations and practiced responding dialectically (e.g., “I see your point of view even though I do not agree with it”). Given the abbreviated nature of the PIU DBT treatment, we collaborated with an outpatient team trained to deliver extended DBT programming to offer a step-down care approach to continue patient therapy following their discharge.

### 2.4. Data Analyses

Descriptive and inferential statistical approaches (e.g., count, percent frequency, mean ± standard deviation, paired *t*-test, MANOVA, Spearman correlation) were applied to summarize trends and determine significant differences in staffing, cases of restraint and seclusion, incidence of injury, and patient psychological scores, at admission to the PIU and at discharge from the PIU. All statistical analyses were completed using IBM SPSS Statistics for Windows (Version 26.0). Non-parametric approaches were applied when data did not show a normal distribution. Significance level was set at *p* < 0.05. All data analyses were performed on de-identified data and the key findings are presented based on aggregate data.

## 3. Results

### 3.1. Patient Demographic and Clinical Profile

Table 1 describes the patient population treated at the PIU during the study period (N = 190). Briefly, a majority of the patients identified as female (56.3%), followed by male, transgender, and those preferring not to answer (at 33.2%, 4.7%, and 5.8%, respectively). These patients were mostly White/Caucasian (63.2%), identified as biracial/multi-racial (16.8%), or Black/African American (8.9%). Most of these patients also identified as not Hispanic or Latino (72.1%). The top three psychiatric diagnoses of these patients were Depression (68.4%), followed by Trauma and Stressor-Related Disorders (9.5%), and Disruptive Behavior Disorders (7.9%). These patients were grouped as tweens (10 to 13 years old; 48.4%) and adolescents (14 to 17 years old; 51.6%). Further, approximately half of the patient population (56.8%) indicated that this was their first inpatient mental health admission (Table 2). Hospitalization for most of these cases lasted between 6 and 9 days (39.0%), or approximately 0 to 5 days (37.0%) (Table 2). Notably, there were fewer than 5% of cases that were hospitalized for an extended length of time (i.e., defined as longer than 20 days) (Table 2).

### 3.2. Patient Psychological Outcomes (PROMIS Scores)

Depressive scores at discharge were observed to be significantly lower than those at admission (59.3 ± 10.5 vs. 65.7 ± 10.0; t (148) = 8.2, *p* < 0.001, *d* = 0.68). A similar trend was also observed for anxiety scores (56.2 ± 11.7 at discharge vs. 60.1 ± 12.2 at admissions; t (149) = 4.5, *p* < 0.001, *d* = 0.37) and anger scores (51.8 ± 12.0 at discharge vs. 55.7 ± 11.9 at admission; t (148) = 4.2, *p* < 0.001, *d* = 0.34). Interestingly, the clinical significance cut-off value for these domains is set at 60 [28]. Thus, our data indicate that while the average scores for depression, anxiety, and anger were clinically elevated at admission (>60), these dipped below the clinical threshold at discharge. Collectively, these trends indicated a marked improvement in depression, anxiety, and anger symptoms among patients resulting from their admission to the PIU at the study site. However, our analysis did not show any significant association between PROMIS scores and length of hospitalization (*F* (12,320) = 1.12, *p* = 0.34; Wilk’s Λ = 0.89, partial η^2^ = 0.04). This suggests that the length of stay at the PIU may not necessarily influence the extent of symptom change or benefit that the patient received from the program. Patients provided positive feedback toward their treatment at the PIU, whereby a majority indicated receiving “excellent” or “good” care (39.6% and 45.8%, respectively), and that they “generally” (44.8%) or “definitely” (40.1%) received the type of help they needed.

### 3.3. Trends of Staff Injury at the PIU

During the study period, there were 129 instances of staff injuries caused by 41 patients (52.8% identified as male patients and 47.2% female). On average, these patients were 13.5 years old, 158.6 cm in height, 73.4 kg in weight, and had a BMI of 26.7. Our analyses indicated that the incidences of staff injury were not significantly correlated to patients’ age (r = 0.47; *p* = 0.20), height (r = 0.03; *p* = 0.95), weight (r = −0.16; *p* = 0.71), or BMI (r = 0.06; *p* = 0.70). Further, we observed that staff member injury tended to occur the most during the mid-afternoon (75.0% of reported total injuries) than other shifts. However, our data did not indicate significant differences in daily staff-to-patient ratio during this shift (15:00–23:00; all *p*-values above 0.05). In contrast, data reflecting the evening shift (19:00 to 07:00) noted higher incidences of staff injury on days when there were fewer staff managing patients. Over the course of this study, we showed that the usage of restraint and seclusion decreased slightly (13.8 h at the beginning of the year to 11.1 h at the end of the year). This difference was not statistically significant (*p* = 0.24). However, the use of restraint and seclusion was significantly associated with an occurrence of staff injury (χ^2^ = 17.4; *p* < 0.001). There was a fewer number of extensive staff injuries requiring reporting to the Occupation Safety and Health Administration (OSHA) when restraint and seclusion were not used to manage aggressive patient behavior than when it was used (12 incidences of staff injury vs. 14 incidences, respectively); but the difference was not statistically significant.

## 4. Discussion

The current study evaluated the effect of interdisciplinary PIU programming involving therapeutic groups that incorporated DBT material, medication management, and family therapy for adolescents between the ages of 12 to 17 years. We assessed patient outcomes via self-report measures of depression, anxiety, and anger at the beginning and end of their PIU admissions. Statistically significant decreases in patient-reported symptoms occurred at the end of the hospitalization. Further, analyses of pertinent variables associated with contributors to staff member injury indicated that specific periods of the day were associated with a higher likelihood of staff injury and that injuries occurred more often when restraint or seclusion were applied to manage patients’ aggressive behavior. 

The care received at this short-term PIU resulted in positive patient outcomes. Patients self-reported symptoms of depression, anxiety, and anger significantly reduced at the end of treatment. This PIU delivered DBT-based group therapy along with individualized family therapy and medication management. Our observation is consistent with the outcomes of another research that has documented the benefit of this type of therapy [13]. However, a lack of a comparison group before the implementation of this model of care represents a limitation of the current study. The current research team began conducting standard outcome measures upon admission and discharge from the unit to permit the evaluation of treatment outcomes [33]. Outcome measurement was not a standard part of practice at this inpatient unit beforehand. Due to this, we recommend that other inpatient programs incorporate outcome measurement into psychiatric care from the outset to facilitate outcome studies. We believe this will help with establishing best practices and other clinical guidelines that will improve patient care and clinical effectiveness [34].

While we observed positive patient outcomes from our PIU, we did not note statistically significant difference in the number of staff injuries over the study period. However, the use of restraint and seclusion was correlated to the incidence of minor and major staff member injuries, whereby injuries occurred more often when this technique was used. This is consistent with the findings from another study [19]. Interestingly, though major injuries (i.e., OSHA reportable) alone did not seem related to the use of restraint or seclusion to manage aggressive behavioral issues. These major injuries occurred equally when managing aggressive behaviors with or without the use of this technique. Notably, we documented a higher occurrence of injury during the overnight hours when less staff were present to manage patients. During these hours, there are typically no structured programming tasks, as patients spend time with their families, participate in leisure activities, and prepare for sleep. Based on our findings, we recommend that PIUs introduce more structured activities for staff to implement for patients who have difficulty sleeping or struggling during leisure time. Additionally, for aggressive patients who tend to stay awake at night, unit leadership should consider adding extra staff to enhance the safety of staff members.

Incidences of staff injuries could potentially be lowered by applying structured behavioral treatment plans, rather than using restraint and seclusion [19,35]. At our study site, we typically alternated between less and more preferred activities to encourage safe behavior and participation. However, individualized treatment programs may be necessary for patients at risk of injuring others. For instance, one research team demonstrated that conducting individualized behavioral assessments to create personalized treatment programs led to positive outcomes for youth while it concurrently decreased incidences of staff injuries [17,19]. Future research should explore whether adding individualized behavior plans can further reduce the use of restraint and seclusion that could subsequently lead to decreasing the number of staff injuries in PIU care settings.

The current study has some limitations. First, the outcome measures were not collected before implementing the new DBT programming, which would have been useful for tracking and comparing symptoms before and after PIU admission. Due to the retrospective nature of our analysis, we were unable to determine the relative impact of DBT programming on patient outcomes compared to other clinical interventions such as medication management, the structured hospital environment, and family therapy. Future research should address these issues through prospective studies to better understand the benefits of PIUs for adolescents and their families. Second, we could not administer health outcome measures that evaluate the maintenance of treatment effects post-discharge. Third, due to the acute nature of the inpatient unit, DBT was primarily implemented in a group setting focused on skills. Additional components of DBT, such as individual therapy, were not standardized but could have provided further benefits.

## 5. Conclusions

The key findings from this study indicate that the clinical care provided at the PIU at the study site significantly reduced symptoms of depression, anxiety, and anger in admitted patients. These results align with previous studies demonstrating the benefits of a structured environment and DBT programming. However, despite the evidence-based care, some patients continued to exhibit aggressive behaviors that resulted in staff injuries. We identified environmental factors associated with these injuries, including the time of day and the use of restraint and seclusion to manage aggressive behaviors.

### Implications

Patients requiring psychiatric inpatient care represent a highly challenging population that demands considerable resources for effective treatment. Despite this recognition, there is still a lack of research on how to effectively work with this population and anticipating the expected patient outcomes. This study provides patient outcome data demonstrating the effectiveness of a clinical care model that includes DBT and other healthcare services. Additionally, it offers descriptive information about the conditions under which staff injuries occur. The PIU setting documented a higher number of staff injuries, and the restraint and seclusion technique was applied, as well as during evening and overnight shift hours. Researchers and practitioners can replicate these analyses to identify when and how staff injuries occur, and develop appropriate interventions for their respective clinical care settings.

## Figures and Tables

**Table 1:** Demographic characteristics of the patient population at the PIU of the study site during the study period.

Characteristic	n	%
Primary Admission Dx		
Depression	130	68.42
Anxiety	13	6.84
Disruptive Behavior Disorder	15	7.89
Bipolar and Related Disorder	8	4.21
Trauma and Stressor-Related Disorders	18	9.47
Schizophrenia Spectrum Disorders	3	1.58
Eating Disorders	1	0.53
Other Dx	2	1.05
Total	190	100
Gender Identity		
Female	107	56.3
Male	63	33.2
Transgender	9	4.7
Prefer Not to Answer	11	5.8
Total	190	100
Race		
American Indian or Alaska Native	6	3.2
Asian	3	1.6
Black or African American	17	8.9
White	120	63.2
More Than One Race	32	16.8
Prefer Not to Answer	12	6.3
Total	190	100
Ethnicity		
Hispanic or Latino	37	19.5
Not Hispanic or Latino	137	72.1
Prefer Not to Answer	16	8.4
Total	190	100
Age Group		
TPU (10–13 Years)	91	48.43
APU (14–17 Years)	97	51.6
Total	188	100

**Table 2:** Admission characteristics of patient population at the PIU of the study site during the study period.

Characteristic	n	%
Admission Number		
1	108	56.8
2	27	14.2
3	11	5.8
4	15	7.9
5	14	7.4
6	2	1.1
7	5	2.6
8	2	1.1
9	3	1.6
10+	3	1.6
Total	190	100
Length of Stay (Days)		
0–5	57	37.01
6–9	60	38.96
10–14	18	11.69
15–19	12	7.79
20+	7	4.55
Total	154	100

## Data Availability

The data that support the findings of this study are not publicly available due to privacy and legal restrictions at the study site. However, it can be made available from the authors upon reasonable request and with permission from the healthcare organization from which the data were collected.
